# Periscope of Dermatology

**Published:** 1896-03-28

**Authors:** 


### Periscope of Dermatology.

**Psoriasis.**—Bulkley[1] considers that psoriasis is a constitutional, rather than a local malady, and draws his deductions from an analysis of 366 cases. He considers that physicians are too often satisfied with simply removing the eruption, without considering, and trying to cure the underlying cause. He finds that, with regard to frequency, the disease appears in about 4 per cent. of all skin disorders, and belongs to the early period of life. While heredity has relatively little importance, the evidence increases that psoriasis is more or less closely allied to the blood states known as gouty and rheumatic. In many cases the urine is hyper-acid, and contains deposits of urates, oxalates of lime, &c. Attention to the condition of the urine is of great importance in the treatment of cases where the eruption is of a congested variety.

Jamieson,[2] in a clinical lecture, also ascribes an early age for the commencement of the disease. With regard to treatment, he recommends the application of salicylic vaseline to remove the horny accumulations, to be followed—when the skin is free from scales—by the application of the following ointment: ℞. chrysorobin, grs. 25; salicylic acid, grs. 10; ichthyol, ♏. 25; green soap, ʒi.; and vaseline, ℥i. He does not consider that the administration of arsenic can be regarded as curative, but states that the greatest benefits are gained by its means in first attacks, in psoriasis of the head, and in constitutional cases. He gives a warning against the use of thyroid extract, unless the patient is under the care of a trained nurse or in hospital.

Pearse[3] describes a condition which he names gouty psoriasis, differing from the ordinary disease in that the eruption is not confined to the extensor surfaces, and is mingled with eczema; moreover, it does not commence in early life, but attacks gouty middle-aged persons. He finds that the condition does not yield to, but is rather aggravated by ordinary remedies, being cured by the ordinary gouty treatment, together with attention to diet, &c. It is not certain whether this condition is not in reality eczema.

Hemsted[4] mentions a case of psoriasis of twenty-six years' duration, which was cured after taking arsenic for about five weeks. The patient suffered from one attack of arsenical poisoning during the treatment. Locally mercurial applications were used, but the writer does not consider that these had anything to do with the cure.

Hutchinson[5] showed a man, aged seventy, with an ordinary eruption of psoriasis on the elbows, palms, and finger ends, but rupia-like crusts existed on the forearms. The knees and the rest of the body were free. Although resembling syphilis, there was no evidence of the disease.

Wolf[6] reports a case of psoriasis of three years' duration, which was treated with a bath of tar vapour. The bath was given in an ordinary bath cabinet, and the body was subjected to the vapour for fifteen minutes; after the bath the skin was wiped dry and rubbed with alcohol. Great benefit was derived from the treatment, which however is only recommended for diffuse cases.

Cantrell[7] recommends the internal administration of oil of copaiba in psoriasis, which he gives in three-minim doses three times a day. A complete cure followed in two cases after six weeks' treatment.

**Acne.**—Colcott Fox[8] reports some cases of acne scrofulosorum which occurred in infants. The eruption consisted of indolent, small papulo-pustules, disseminated, for the most part, sparsely, without definite grouping, and situated most frequently on the external aspects of the limbs. The papules appeared about hair follicles, and after becoming pustular dried up, but left central plugs which, in their turn, shelled out, leaving a crater. Faint scars were left after the eruption. No strong history of tubercle was given in these cases, and the author does not regard the affection as directly tuberculous, but as one which may occur in any child of poor nutrition.

Dale James[9] relates the case of a boy, aged 13, who suffered from paraffin acne. The boy's occupation consisted in winding iron wire which had been hardened in a paraffin bath, and his clothes, especially the trousers, became saturated with oil. The eruption was confined almost entirely to parts where the friction of the clothes was pretty constant, and consisted of a series of black plugs in dilated follicles, round which were erythematous zones; in some cases fleshy exuberances projected from the openings of the follicles. The eruption disappeared without any definite treatment after rest in bed, and another boy, doing the same work, suffered in the same way, but cured himself by washing in soap and water.

Boeck[10] draws attention to a new property of whale oil. Its peculiar power of penetration is well known, but the writer states that it restrains the vitality and growth of bacteria in the skin. He therefore recommends its use in acne vulgaris.

**Pemphigus.**—Pernet[11] records four fatal cases of acute pemphigus occurring in young journeymen butchers. He considers that the disease is due to a micro-organism, which gains entrance by a wound on the hand or fingers. He therefore suggests that wounds on the fingers and whitlows, in butchers and others handling meat, should be thoroughly disinfected with powerful antiseptics.

Kaposi[12] considers that pemphigus is a purely clinical idea, dependent neither on morphological nor on histological characters. He would still use the term pemphigus for most cases of dermatitis herpetiformis. Rosenthal,[13] however, considers that the term pemphigus designates no morbid entity, but a definite elementary form of skin eruption. He therefore applies the name to every bullous eruption.

**Erysipelas.**—Straus[14] gives the results of the treatment of erysipelas by hypodermic injections of anti-streptococcal serum. The best results were obtained when a strong serum was used, and the mortality was very low. The injection was followed by a subsidence of the inflammatory phenomena, and by a great improvement in the general condition. Relapse and suppuration are rare after this treatment. The usual dose required to cure erysipelas varies from 20 to 40 cubic centimetres.

Allen[15] insists on the ætiological relationship of erysipelas to some existing dermatosis, and found out of one hundred cases just one-half were traceable to some existing skin lesion. With regard to treatment, he recommends the application of bands of rubber adhesive plaister in such a way as to entirely enclose the inflamed area, followed by the painting of the whole enclosed area, together with apparently healthy skin, with a 10 to 50 per cent. solution of ichthyol in collodion.

**Lupus.**—Baum,[16] in a clinical lecture on lupus, recommends the following treatment for ulcerating lupus where other operative measures are not advisable. A 10 per cent. ichthyol ointment is first applied, followed by a 10 per cent. aristol ointment; the following ointment is finally used: ℞: alumnol 20 grs., lanolin grs. 10, and vaseline 70 grs.

Besnier[17] states that out of hundreds of cases of lupus which accidentally contract erysipelas, in very few is the disease cured, and if apparently benefited it will relapse. He considers that erysipelas only produces a temporary inhibition of the disease, and so condemns the mode of treatment of lupus by inoculation of erysipelas as unjustifiable.

Smyth[18] records the good results to the appearance of a patient, whose nose and upper lip had been destroyed by lupus, obtainedby the use of a mask consisting of an artificial nose and upper lip bearing a moustache; the whole mask was kept in position by means of a spectacle frame.

Morris[19] relates a case of lupus vulgaris, which had been treated for nineteen months with thyroid extract. When the treatment was commenced, the disease was making rapid progress, but now all ulceration had stopped, and the disease was steadily improving. Morris[20] also describes a case of lupus erythematosus where the erruption had occurred at the site of the scars left by mosquito bites.

**Parasitic Diseases.—Tinea.**—Mibelli,[21] having repeated the experimets of Saborand, confirms the doctrine of the plurality of the trichophyton. He was, however, unable to meet with the disease described as ringworm with small spores. He considers that the division between animal and human ringworm is unfounded, since he believes that a trichophyton may pass from man to horse and *vice versa*.

Adamson[22] recommend the following method for the preparation of permanent stained specimens of ringworm fungus. The hair or scales are placed on a slide with a drop of a 5 to 10 per cent. solution of caustic potash and a cover glass applied; the preparation is then allowed to remain for half-an-hour to clear. The specimen is then hardened by washing, under the cover glass, with a 15 per cent. solution of alcohol, and the cover glass is removed. The slide or cover glass is then dried in a flame, and the specimen stained by the Gram-Wiegert method. After decolorising in anilin oil the specimen is mounted in Canada balsam.

Under the name plica polonica Ohman-Damesnil[23] refers to a matting of the hair in an extricable mass, accompanied by exudation of glairy fluid from the scalp and due to pediculi. Entire removal of the matted mass is necessary before any treatment can be employed.

**Tumours, &c.**—Leslie Roberts[24] reports a case of mycosis fungoides which occurred in an old woman. The first appearance of the disease consisted of a patch of moist eruption which had begun four years previously. During the past year numerous small tumours appeared, and the whole surface of the skin became affected with desquamating dermatitis. The patient suffered from a hectic temperature, and after death a tubercular cavity was found in the lung. He suggests that the preliminary eruptions in these cases are not dermatitis, but are veritable intradermic neoplasms. He concludes, after careful histological notes, that at present there is no evidence pointing to the microbic origin of the disease.

Jamieson,[25] in a paper "On some Superficial Affections of the Red Portion of the Lips," describes an ulceration of the lower lip which, though resembling eczema, after histological examination appeared to be related to epithelioma, since there was down-growth of the strata malphigi and infiltration of the dermis with leucocytes. The case, however, recovered after cauterization with a 50 per cent. solution of lactic acid followed by a zinc ichthyol dressing.

Dubreuith[26] discusses the occurrence of warts on the sole of the foot. He finds them to be most common beneath the head of the third metatarsal bone, but they are also found on the heads of the first and fifth metatarsal bones and under the heel. These are the points of support of the arch of the foot, and are uncommon places in which to find ordinary corns. These warts are affected by the atmosphere in the same way as corns; curetting, after local anæsthesia produced by cocaine, is the most rapid and radical method of treatment. A careful microscopical investigation is also to be found in the paper.

**Angio-Neurotic Œdema.**—Nammack[27] relates the case of a man, aged 35 years, who during five years suffered from attacks of urticaria, which resisted diatetic and other treatment. Hypodermic injections of pilocarpine acted like a charm, and this, too, when all the dietetic restrictions were removed. There was no neuropathic taint in this case, but the patient was suffering from overwork and mental strain. The author considers that the disease is a vaso-motor neurosis, and so the pilocarpine acts by relaxing the spasm of the vessels, upon which the resulting exudation depends.

**Dermatitis Repens.**—Stowers[28] describes an example of this rare disease which affected an old woman, aged 67 years. The disease had commenced in her thirtieth year, about fourteen days after her second confinement, by suppuration under the right thumb nail. The nail was removed, but the wound never healed. The other nails of both hands and feet became similarly affected and destroyed. Considerable swelling and ulceration was also present in some of the fingers. The general organs were healthy. She died with some symptoms of abdominal cancer, but no post-mortem examination was made.

**Pityriasis rubra.**—Skaife[29] reports the occurrence of the disease in a woman aged 59 years, who was being treated for varicose ulcer of the leg. After the application of sulphate of zinc solution to the ulcer a marked redness was noticed on the skin round the ulcer. This condition rapidly advanced over the whole body, and became general within thirty-six hours. The skin became covered with thin wafery scales, but beneath them was of a bright scarlet colour. The eruption lasted for several months, and just before it disappeared the patient suffered from diarrhœa and smoky urine. She ultimately made a complete recovery.

**General.**—Walter Smith,[30] in an article on "Diet in the Œtiology and Treatment of Diseases of the Skin," states that diet may influence the skin in four ways—(1) through the general nutrition of the body, (2) by acting as a reflex stimulus from the gastro-intestinal surfaces; (3) by the absorption into the blood of irritating substances, or of chemical products; and (4) by becoming one of the channels of elimination of effete materials. He, however, sums up by stating that very few skin diseases are directly traceable to dietetic errors, and such as are, are of a transitory nature. Diet, too, has little influence in curing any disease of the skin.

Pye Smith[31] refers to four forms of skin eruptions which occur in the course of Bright's disease—(1) A bright diffused rash, most marked on the trunk; (2) an eruption of large discrete red papules most frequently seen on the outer aspects of the thighs and arms; (3) a moist dermatitis, resembling eczema, but not affecting the flexures of the limbs; and (4) an extensive exfoliative dermatitis, resembling universal exfoliative dermatitis. None of these affections, with the exception of erythema leve, are at all common.

Mackenzie,[32] in a lecture "On the Advantages to be Derived from the Study of Dermatology," points out that general eruptions may be produced by the following factors—(1) Heredity or diatheses, (2) by the influence of the nervous system, (3) by diet, and (4) by specificity. Several examples of diseases, traceable to each group, are detailed.

[1] Med. Press, Nov. 27, 1895, p. 547; Ed. Med. Journ., Dec, 1895, p. 571. [2] Clin. Journ., Jan. 1, 1896, p. 14. [3] Lancet, Dec 14, 1895. [4] B.M.J., Dec. 28, 1895 [5] Clin. Journ., Nov. 6, 1895, p. 28. [6] Med. Record, Jan. 11, 1896. [7] Brit. Journ. of Derm., Jan. 1896, p. 96. [8] Brit. Journ. of Derm., Nov., 1895, p. 341. [9] Quarter. Med. Journ., Jan., 1896, p. 151. [10] Ed. Med. Journ., Jan., 1896,, p. 668. [11] Brit. Med. Jour., Dec. 21, 1895, p. 1,554. [12] Brit. Journ. of Derm., Nov., 1895, p. 359. [13] Brit. Journ. of Derm., Nov., 1895, p. 360. [14] Brit. Med. Journ., Jan. 11, 1896, p. 109. [15] Med. Record, Nov. 23, 1895, p. 723. [16] Int. Clinics, IV. 2, p. 347. [17] Med. Week., Dec. 27, 1895. [18] Lancet, Jan. 25, 1896, p. 229. [19] Brit. Journ. of Derm., Dec., 1895, p. 393. [20] Brit. Journ. of Derm., Jan. 1896, p. 17. [21] Brit. Journ. of Derm., Dec., 1895, p. 383. [22] Brit. Journ. of Derm., Dec., 1895, p. 373. [23] Int. Clinics, IV. 2, p. 340. [24] Lancet, Nov. 23, 1895, p. 1,283. [25] Brit. Med. Journ., Dec. 7, 1895, p. 1,410. [26] Ed. Med. Journ., Dec., 1895, p. 570. [27] New York Med. Rec., Jan. 4, 1896. [28] Brit. Journ. of Derm., Jan., 1896, p. 1. [29] Lancet, Nov. 2, 1895, p. 1,107. [30] Dublin Journ., Nov., 1895, p. 377; B. M. J., Nov. 30, 1895, p. 1,353. [31] Brit. Med. Journ., Nov. 30, 1895, p. 1,349. [32] Brit. Med. Journ., Jan. 25, 1896, p. 193.

